# Series: Public engagement with research. Part 4: Maximising the benefits of involving the public in research implementation

**DOI:** 10.1080/13814788.2023.2243037

**Published:** 2023-08-23

**Authors:** Laura Swaithes, Laura Campbell, Sibyl Anthierens, Magdalena Skrybant, Dieuwke Schiphof, Helen French, Maarten de Wit, Steven Blackburn, Krysia Dziedzic

**Affiliations:** a Impact Accelerator Unit, Versus Arthritis Primary Care Centre, School of Medicine, Keele University, Staffordshire, United Kingdom; b Department of Family Medicine and Population Health, University of Antwerpen, Antwerpen, Belgium; c National Institute of Applied Health Research Applied Research Collaboration West Midlands, Institute of Applied Health, University of Birmingham, Edgbaston, United Kingdom; d Department of General Practice, Erasmus MC University Medical Center, Rotterdam, The Netherlands; e School of Physiotherapy, Royal College of Surgeons in Ireland University of Medicine and Health Sciences, Dublin, Ireland; f Patient Research Partner, Stichting Tools, The Netherlands; g Institute of Applied Health Research, University of Birmingham, Birmingham, United Kingdom

**Keywords:** Patient and public involvement, implementation, general practice, knowledge mobilisation

## Abstract

This final article in the four-part series focuses on the often neglected yet important role of the public in implementing research in General Practice and Primary Care more broadly. Experience in implementation of findings from research with public engagement in Primary Care has highlighted how partnership working with patients and the public is important in transitioning from ‘what we know’ from the evidence-base to ‘what we do’ in practice. Factors related to Primary Care research that make public engagement important are highlighted e.g. implementing complex interventions, implementing interventions that increase health equity, implementing interventions in countries with different primary healthcare system strengths. Involvement of patients and public can enhance the development of modelling and simulation included in studies on systems modelling for improving health services. We draw on the emerging evidence base to describe public engagement in implementation and offer some guiding principles for engaging with the public in the implementation in General Practice and Primary Care in general. Illustrative case studies are included to support others wishing to offer meaningful engagement in implementing research evidence.

KEY MESSAGES

- Patients and the public are essential partners for taking evidence into General Practice services.
- This paper presents practical guiding principles to help General Practice stakeholders to navigate the complexity of implementation with the public as central partners
- Understanding local context and building and sustaining meaningful relationships are critical for optimising public involvement in implementation

## Introduction

Public engagement, as an important requirement in health and care research, has been adopted in major national research organisations and initiatives such as the National Institute for Health and Care Research, UK, and Co-Act in Europe, which also describe public engagement in the dissemination and implementation of research in the later stages of the research cycle [1,2]. An increased emphasis on reporting public engagement has resulted in a growing evidence base on how it can be embedded throughout the research cycle [3]. Implementation, where research evidence is embedded into everyday practice [4], is notoriously messy and is often a neglected stage of the research cycle. The complexities associated with getting evidence into practice mean that public engagement in implementation is a relatively novel and less developed area. Many factors related to Primary Care research make public involvement and engagement important e.g. implementing complex interventions, implementing interventions that target health equity, implementing interventions in countries with different primary healthcare system strength. Effective public engagement in implementation can help to ensure that research evidence (what we know) is translated into practice (what we do) quickly and successfully, ensuring high-quality services for all [5]. Involvement of patients and public can enhance the development of modelling and simulation included in studies on systems modelling for improving health services. However, implementation is not straightforward, and all stakeholders will likely to encounter challenges. Despite this, the benefits of having public contributors central to implementation projects far outweigh the challenges [6]. Public engagement in implementation can be considered as embedded as public engagement in every other stage of the research process. This paper draws on real-world examples to develop key principles for how partnership working with public contributors can be effective in implementation. Practical examples demonstrate how public contributors can be involved in research implementation in the General Practice setting despite the different local healthcare settings.

Implementation is a practical process which draws upon a range of strategies or methods to embed knowledge (in the form of useable innovations) into clinical practice to improve outcomes and quality of care [7]. Implementation is important in reducing research waste and the delay in adopting evidence into practice, known as the ‘evidence-to-practice gap’. An increasing body of implementation literature seeks to address the evidence-to-practice gap, yet there are a plethora of theories, models, frameworks and terminology to navigate [8]. Box 1 provides an overview of key terms used in implementation, illustrated with a practical example of the cough consultation in Primary Care [9].

Challenges in implementing new ways of working include a lack of a good evidence base of effective implementation strategies [10], workforce issues (e.g. attitudes, skills, knowledge, and resistance to change) [11,12], and conflicting drivers and agendas between different stakeholders. All these factors impact on the involvement of public contributors in implementation. A key issue is identifying and agreeing on who is responsible for implementation of research results. It may not be the researcher’s primary responsibility but a knowledge mobilisation practitioner can be perfectly placed to broker research knowledge across boundaries as ‘boundary spanners.’

**Figure 1.**
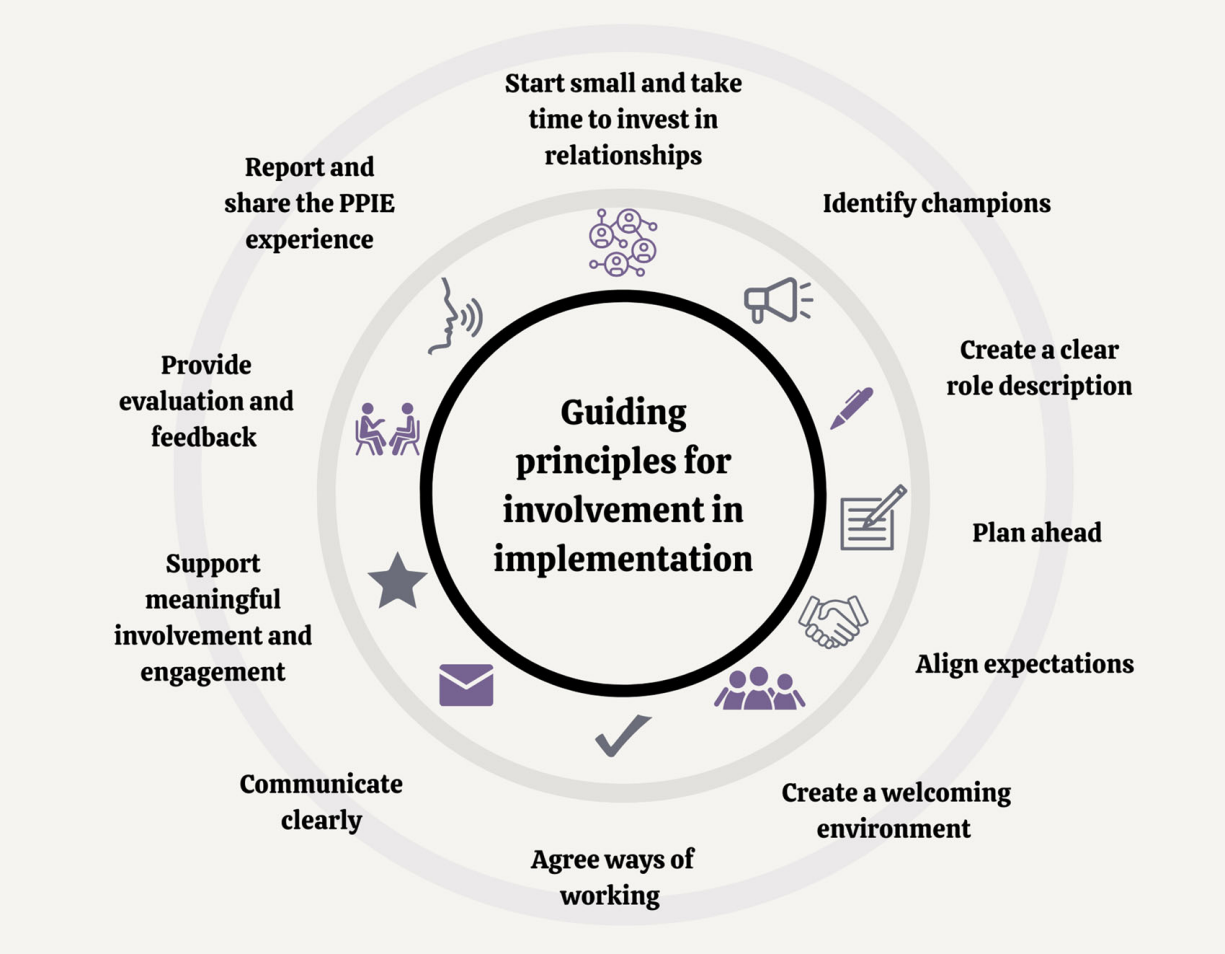
Guiding principles for working effectively with patients and the public to implement research into General Practice.

Limited guidance or resources are available to guide implementation stakeholders on how best to involve public contributors as implementers of research in General Practice. As a result, involving public contributors in implementation projects can often be tokenistic with some concerned that public involvement and engagement can increase project costs and that patients or the public might have biased views on specific health issues [13]. Key principles to help to embed public contribution in implementation are proposed in Figure 1.

Patient and public networks have been described as an ‘untapped resource’ when implementing research evidence [5]. We have previously demonstrated the emerging role of public contributors as implicit facilitators for mobilising knowledge in implementation practice [14]. Alongside health professionals, they play an important role in optimising implementation and are appropriately positioned to broker and influence both the producers and users of research knowledge [5,15]. Public contributors, therefore, can and should have a role in implementing research evidence to ensure that findings are ready and useable for uptake in Primary Care practice.

## Context

Within Primary Care, public contributors are important stakeholders uniquely positioned (as both contributors to and end users of best evidence) to build relationships and better understand local contexts to take opportunities beyond the research into real-world General Practice. A review of reviews on the evidence-to-practice gap in Primary Care identified several effective strategies for closing this gap [16]. Earlier work identified how public contributors generated innovative ideas and solutions to local service problems and catalysed broader change [17]. As implementers, public contributors can help to ensure that end-users have access to information which can lead to a more equal balance of power and better understanding for making decisions regarding their care [14]. However, there is a limited evidence-base to draw on what this looks like and questions remain regarding the role and support required for public involvement and engagement to make meaningful contributions to implementation [10,18].

To optimise public involvement and engagement in implementation and mobilisation of knowledge in General Practice, understanding context and building and sustaining relationships require careful consideration from the outset. Context is a broad concept that concerns ‘weaving together’ of evidence-based practice (e.g. an innovation) with a team, department or organisation [19]. Implementation requires acknowledgement of real-world conditions rather than controlling for them [8], which can help identify strategies to mitigate potential implementation challenges and maximise uptake. For example, a local practitioner will have a good overview of context-specific barriers in their own (working) environment, healthcare setting or local area and, alongside patients and the public, will understand local population needs and context. The context of Primary Care, and more specifically General Practice, will vary between countries and each setting may have a network of public contributors to draw upon (e.g. in the UK many General Practices have a Public Participation Group that work with a range of healthcare professionals to involve the patient voice in shaping services).

Implementation of an innovation, a new way of working, or system change is enhanced by strong relationships and the involvement of all relevant stakeholders, including public engagement, at the outset [20]. Not only is this due to the previously mentioned reasons regarding understanding of context, but building relationships with public contributors and their networks can help to inform decisions and provide support for long-term sustainability. It is also important to recognise that implementation is not a single activity at the end of a project in a linear, staged approach. Implementation is a complex, relational, context-driven approach that requires consideration and planning throughout the research cycle from the outset [21].

The following section outlines examples of how public engagement in implementation has been effective and how public engagement has added value and benefitted an implementation project.

## Guiding principles for involving patients and the public in implementation

There is a growing but limited body of literature involving patients and the public in implementation. Yet there are few examples where public involvement has been embedded successfully in implementation in General Practice. This section outlines key guiding principles that can enhance effective involvement of patients and the public to successfully move the benefits of research and other evidence-based practices into Primary Care practice (illustrated in Figure 1). Principles are based upon the work of the Keele University Link group: a knowledge mobilisation public contributor group, that grew from Keele’s patient and public involvement and engagement group [22]. This is described further under ‘evaluation’ in the Case Study following.

It is unlikely to need all the components outlined in Figure 1 to succeed. These guiding principles illustrate what can work in practice (e.g. [23]). Table 1 provides a detailed description of these guiding principles, and the following section demonstrates the practical application of public involvement and engagement in implementation using a completed case study in General Practice as an example.

**Table 1.** Guiding principles for involving patient and public contributors in implementing research.

1	Start small and take time to invest in relationships	Start small and early and allow time for recruiting and setting up a public involvement group. Encourage public contributors to be involved in the implementation of research by firstly building relationships with people who would have a personal investment in the research output – for example they have direct or indirect experience of the health condition or are involved with a charity who would benefit from the innovation. Consider recruiting people from a range of backgrounds or with different experiences: there is richness in diversity.
2	Identify champions	Engaging early adopters to be clinical, academic, or public champions can be beneficial as they can spread the word to other individuals and groups. A stakeholder mapping exercise may be helpful in identifying key people, relationships, and networks (an example can be found in Supplementary Figure S1)
3	Create a clear role description	Think about *who* you can involve, *what* knowledge and experiences are important to the implementation phase of your project, and *how* working in partnership with public contributors can add real value to your project. A complement of diverse skills, knowledge and experience is beneficial in defining your role(s).
4	Plan ahead	Ensure funding for public engagement time has been accounted for. (e.g. https://www.hra.nhs.uk/about-us/news-updates/new-guidance-easier-payment-public-research-contributors/). Involving members of the public can be time consuming and can create unexpected diversions to the plans you had in your mind. Factor this into your overall project plan, be flexible and allow room for potential change.
5	Align expectations	Set aside time to discuss public involvement in implementation and reach a shared understanding with everyone in the team about expectations, from both sides.
6	Create a welcoming environment	Create a non-hierarchal environment in which everybody can express their views, feels that their opinions have been listened to and acted upon, and can contribute to shared decision-making. Allow plenty of time for team members to get to know each other and be comfortable in expressing their ideas and opinions e.g., starting meetings with ‘icebreaker’ activities.
7	Agree ways of working	This might be a formal ‘Terms of Reference’, but it might be a more informal list of agreed approaches. Everyone must agree to these, and it can be helpful to remind people of these at the start of meetings. Be flexible and adaptable
8	Communicate clearly	It is important to encourage all team members to avoid jargon/acronyms and present information clearly. Developing a glossary and an ‘acronym buster’ for all team members to use is a helpful way to clarify any scientific or medical terms identified by the panel to be unfamiliar to them.
9	Support meaningful involvement and engagement	Co-design and co-production principles can help engage all team members collaboratively and ensure equity by supporting a culture of ‘working together’ and ‘shared decision making’ [34].
10	Provide evaluation and feedback	Set aside time to incorporate regular evaluation of the impact of public engagement on the implementation process and provide opportunities for public engagement members to provide and receive feedback on their role to achieving implementation objectives. Evaluation of the impact of public engagement on implementation should take place throughout the project and not just at the end.
11	Report and share the public engagement experience	Identify opportunities to share and disseminate the involvement of patients and/or the public in implementation to relevant target audiences in collaboration with public engagement. The Guidance for Reporting involvement of Patients and the Public (GRIPP2) checklist, an international framework for reporting public engagement should be considered at the start of any project [3]

## Case study

One example of embedded public engagement in the implementation of innovations in General Practice is the Joint Implementation of Osteoarthritis Guidelines Across Western Europe (JIGSAW-E) project based on findings from the Management of OsteoArthritis In Consultations (MOSAICS) study [23]. JIGSAW-E aimed to implement four key innovations from the MOSAICS study across Primary Care in five European countries:

- A novel osteoarthritis guidebook
- A set of quality indicators
- A model consultation in Primary Care
- Training of health professionals

### Start small, invest in relationships, and identify champions

JIGSAW-E included patients from General Practices from the outset. JIGSAW-E used a cohesive and partnership-focussed Community of Practice (CoP) approach (including patients, clinicians, academics, knowledge mobilisers and project managers) [14] in which public contributors were prioritised and an International Patient Panel was established. Public contributors enjoyed seeing tangible benefits to the communities they represented. In the Netherlands, Patient Research Partners were pleased to see a UK guidebook for osteoarthritis adapted to the Dutch context and actively implemented into practices with GPs and physiotherapists and supported by the Arthritis Foundation.

### Create a clear role description, plan ahead and align expectations

The role of public contributors on the International Patient Panel was jointly agreed by the CoP at the start of the project and specific areas for the role of public champions were clarified. They worked with a Knowledge Broker from Keele’s Link Group and local public engagement coordinators on translating, culturally adapting and disseminating the OA guidebook [24].

Payment was offered for their time throughout the project and all expenses were reimbursed.

### Create a welcoming environment, agree ways of working and communicate clearly

To facilitate strong communication, the Knowledge Broker and public engagement team developed a glossary of terms in plain language and a guide to acronyms. Plain language summaries of the project were provided. Throughout the project, the Knowledge Broker acted as the dedicated point of contact for Public Contributors and all agreed suggestions were acted upon. They were encouraged by the public engagement leads and a Knowledge Broker to get involved in key knowledge mobilisation activities (e.g. [25]). Additional support and time for questions was provided to public contributors between meetings.

### Support meaningful involvement and engagement

Public contributors played a key role in translating complex evidence and recommendations into public-friendly, accessible, and engaging content. Public contributors reminded the team of the importance of creating content in various formats for different demographics and drew on their own varied lived experiences to shape how these were developed.

### Provide evaluation and feedback, and report and share the public engagement experience

Teams from across JIGSAW-E were involved in evaluating public engagement and sharing their experiences.

Evaluation was supported by ongoing feedback from the patient panel during the four-year project and shaped how the public could be involved in how the implementation was conducted.

### Evaluation of impact of public involvement and engagement in implementation

The Keele Link group assessed their transition into implementation. Using qualitative methods (semi-structured interviews followed by a focus group) the experiences of seven implementers (lay members, healthcare professionals (HCPs), academics, researchers) were explored. Findings highlighted that a conducive environment influenced by an established public engagement team promoted knowledge mobilisation. This team was integral to the selection of the public involvement in implementation. Patient and the public adjusted to and understood the differences between research and implementation. Skills of public members were multi-faceted compromising of experience of conditions, life skills, roles in society and networks (an example of these networks is provided in Supplementary Figure S1). Feedback was important to patients and the public in remaining engaged and feeling confident in questioning ways of overcoming barriers to implementation that they may be in a position to influence.

## Implications

JIGSAW-E is an example of well-funded and well-resourced public engagement in implementation, this is not always the case. Table 2 illustrates a spectrum of examples of engagement in implementation, from small scale local projects to larger collaborations. It shows that even with limited resources, much can be achieved through strong partnership working. Importantly, similarities between key features of Table 2 and the literature regarding ‘co’-approaches (co-production, co-design, and co-creation) are noted [26]. For example, priorities for successful co-production centre around creating a shared understanding, identifying and meeting the needs of a diverse group of people, balancing power and voice, developing a sense of ownership, and creating trust and confidence [26]. Similar to co-production, it is important to consider all forms of knowledge (including experience and beliefs, not just research) with the patient voice at the forefront and taking local context into account.

**Table 2.** Description of public involvement in implementation projects and lessons learned.

Name of Project	Description of the Project		Challenges and Enablers to public involvement, and Lessons Learned		
	The context (setting and initial resource)	Nature of public involvement (how many, where from e.g.. charity, advertisement in general practice)	Key challenges/barriers	Key enablers/facilitators	Lessons Learned
JIGSAW-E	The implementation of National Institute for Health and Care Research (NICE) guidelines in UK and European countries [23]	Patients and public members for general practice with joint pain and osteoarthritis recruited by healthcare professions	Differences in healthcare settings; differences in the way in which healthcare is delivered; different levels of empowerment of patients within the general practice setting	A Community of Practice (CoP) approach; flexibility and adaptations of innovations for the context; reimbursement offered; support of a knowledge broker and coordinators	Context of healthcare systems across different countries drives differences in approach so flexibility and a CoP can be powerful facilitators
The Duo Trial:	A pragmatic cluster randomised controlled trial to investigate the additional value of duloxetine as a third-choice medicine in the treatment of hip and/or knee osteoarthritis [35]	Three patients asked by GP to be part of the study throughout. Twice yearly face to face meetings were conducted between the patients and the whole study team with regular communication via email.	Identifying patients willing to be involved Funding the patients due to institutional rules and processes	Liaising with general practitioners for help identifying potential public engagement contributors as they saw relevant patients regularly. Face-to-face meetings twice a year facilitated the relationship and the engagement of the patients with the project team. The same people were involved throughout the project, so no new introductions were needed, which made it possible to stay within reasonable time to discuss the points on the agenda.	Start with a small group if you are inexperienced, invest in building the relationship, make agreements on time, way of contact, rewards and enjoy the extraordinary ideas of laypeople and discuss how they can improve the project.
BeeFree	A project to support better awareness, identification, and management of mental health problems for people with persistent neck and back pain. Collaboration between NHS musculoskeletal and mental health services, Keele University, and charities (Mind mental health charity and the Haywood Foundation). £2,000 initial funding from Q Improvement Lab (Health Foundation) to design a prototype animation for waiting rooms, pilot training event, community of practice, and mental health information repository.	Eight public engagement members (hospital volunteers and patients) were part of the launch event which set out the ambitions for the community of practice. Public engagement members were recruited through existing networks. Public engagement members co-produced initial suite of resources, mapped their own networks for eventual implementation of resources. Public engagement members were key to securing additional £100,000 funding to professionally develop the resources within a bespoke brand and to move the test area out of one hospital service into a broader geographical area, through a commitment to being involved in testing and evaluation of the pilot resources and co-creation of final versions.	Working with a relatively small amount of funding initially as this was needed to develop resources and reimburse public engagement members’ time Conducting meetings online due to Covid meant that public engagement members needed extra support	Involving public engagement members from the start encouraged ownership of final resources and a desire to promote and use them in their volunteer and patient networks Public engagement members were a key part of the community of practice (a group of people coming together who have a like mind to solve problems creatively and collaboratively) and were considered equal partners alongside commissioners, managers, and clinicians. This enabled them to drive forward implementation relevant to their own contexts.	It is possible to use small amounts of funding (in this case £2000) to springboard further funding applications to scale up projects (£100,000 awarded) by including public engagement members at the heart of the project. It is important to develop 2-way, reciprocal, enthusiastic relationships with patients and the public and invest time in these to understand their contexts, drivers and priorities. Knowledge brokering between all stakeholders (clinicians, patients and the public, academics) is important to facilitate co-creation of resources.

Public involvement and engagement in implementation takes time and effort and challenges will be encountered. Planning needs to start early, with time and resources set aside to support stakeholders throughout the ‘messiness’ to have a meaningful contribution.

Public contributors remain an under-utilised partner in facilitating successful implementation of research findings into General Practice. We have taken lessons from public engagement in research and applied our understanding to implementation. Core values for partnership working, such as building and maintaining relationships, sharing power, and, establishing mutual ways of working, can help to ensure that public engagement is embedded throughout the research cycle from priority setting research questions through to implementation. Lessons can be learnt from other fields in which patient experiences are used for implementation in Primary Care e.g. in health promotion, individual patient participation as patient experts in patient care or as participants in education.

## Conclusion

Public engagement in implementation is important in ensuring that ‘what we know’ from the evidence-base is adopted and embedded ‘what we do’ (in practice) quickly and effectively. The guiding principles presented are intended to help those for whom interventions are intended, to become active partners in enhancing the uptake of research in General Practice services. Further work needs to focus on the accurate and clear reporting of the role of public involvement in implementation to build an evidence base to better understand what works, for whom, and in what contexts.

## Supplementary Material

Supplemental Material

## Figures and Tables

**Table:** 

**Key term and description**	**Practical example –** The management of acute cough in Primary Care using an interactive patient booklet as the intervention [9]
**Dissemination:** The active, tailored, and targeted distribution of information or interventions via determined channels using planned strategies to a specific public health or clinical practice audience [27]. Dissemination can be done through different communication channels and technologies, including summaries/briefings, engaging knowledge users in developing and executing dissemination plans, tools creation, and media engagement.	Informative and educational sessions on the use of the patient booklet for acute cough in local general practitioners’ quality circles Informing all GPs by letter about the online e-learning on using the interactive patient booklet for acute cough that is freely available on the national GP website Informative messages on social media aimed at patient communities
**Knowledge translation**: Knowledge translation is ‘the exchange, synthesis, and ethically sound application of research findings with a complex system of relationships among researchers and knowledge users.’ Where knowledge translation differs from implementation science is that knowledge translation does not cover how to implement knowledge [28]. The process of knowledge translation ensures that evidence from research is used by relevant stakeholders in improving health [29]. **Knowledge mobilisation**: In its simplest ‘moving knowledge to where it can be most useful’ [25]. It involves the right people coming together to share and use available knowledge within a given context to solve a problem, improve a service and share best practice. It is transdisciplinary and characterised by heterogeneity of skills, by a preference for transient flatter hierarchies and organisational structures [30]. Unlike knowledge translation, knowledge mobilisation relies more on two-way stakeholder relationships and describes a more multi-directional and ‘messy’ process. It tends to include dissemination, knowledge transfer and knowledge translation in its activities [31]. **Knowledge brokering:** Knowledge brokering is an emerging strategy to promote interaction between research producers and end-users, develop a mutual understanding of goals and cultures, identify issues and problems for which solutions are required, and facilitates the identification, access, assessment, interpretation, and translation of research evidence into local policy and practice [32]. **Implementation science**: ‘The scientific study of methods to promote the systematic uptake of research findings and other evidence-based practices into routine practice’ [33]. Implementation science aims to close the gap between evidence-based practices and the extent to which research findings are integrated into real world settings and practices.	Collaborative and systematic assessment, collation and application of research relating to acute cough is incorporated into a policy brief to advise health authorities on how to safely reduce antibiotics in Primary Care using an interactive patient booklet for acute cough All stakeholders with interest in acute cough to proactively discuss ways in which new knowledge can be shared and adapted to local GP context(s). Patients and public can take responsibility to share knowledge widely by developing lasting relationships with peers, community groups and volunteer groups to encourage use of this knowledge. All stakeholders take and give knowledge around acute cough, learning from each other along the way Working with a ‘middleman’ –who links all stakeholders concerned with acute cough – to develop an interactive patient booklet with agreed aims and objectives for its use in practice. The stakeholders could be patients and the public with experience of acute cough, clinicians with interest or expertise in the area, and academics with research knowledge, as well as IT teams, graphic designers and content creators. The Knowledge Broker, or person in the ‘middleman’ role, can work with all stakeholders to ensure materials are co-produced, with patient experience, ‘on the ground’ clinical know-how and relevant evidence included. A range of formats can be produced dependent on target audience and steer from the public. A Knowledge Broker may also have a role in facilitating discussions regarding the use of the booklet in General Practice (e.g., timing, how to introduce the booklet) **The intervention:** a patient booklet for cough to be used within the cough consultation in Primary Care **Effectiveness research:** looks at whether the patient booklet for cough reduces antibiotic prescribing in Primary Care **Implementation strategies:** the strategies we can employ to help general practitioners to use the booklet within their cough consultation (e.g., educational sessions, trainings) **Main implementation outcomes:** how much General Practitioners use the booklet within the consultation and how much the booklet impacts on antibiotic prescribing.
