# Supplementary material for: Series: Public engagement with research. Part 4: Maximising the benefits of involving the public in research implementation
Source: Eur J Gen Pract. 2023 Aug 23;29(1):2243037. doi: 10.1080/13814788.2023.2243037 (PMC10448833; doi:10.1080/13814788.2023.2243037)

**Supplementary Figure 1. An example of a mapping exercise: Networks, connections and reach of public contributors from the Lay Involvement in Knowledge Mobilisation (LINK) group, Impact Accelerator Unit, Keele University**

The table shows the results of a mapping exercise developed by the Keele patient and public involvement in Knowledge Mobilization group (LINK), with lay members sharing their own contacts and links with a range of different organizations, local, regional, national and in some cases international.


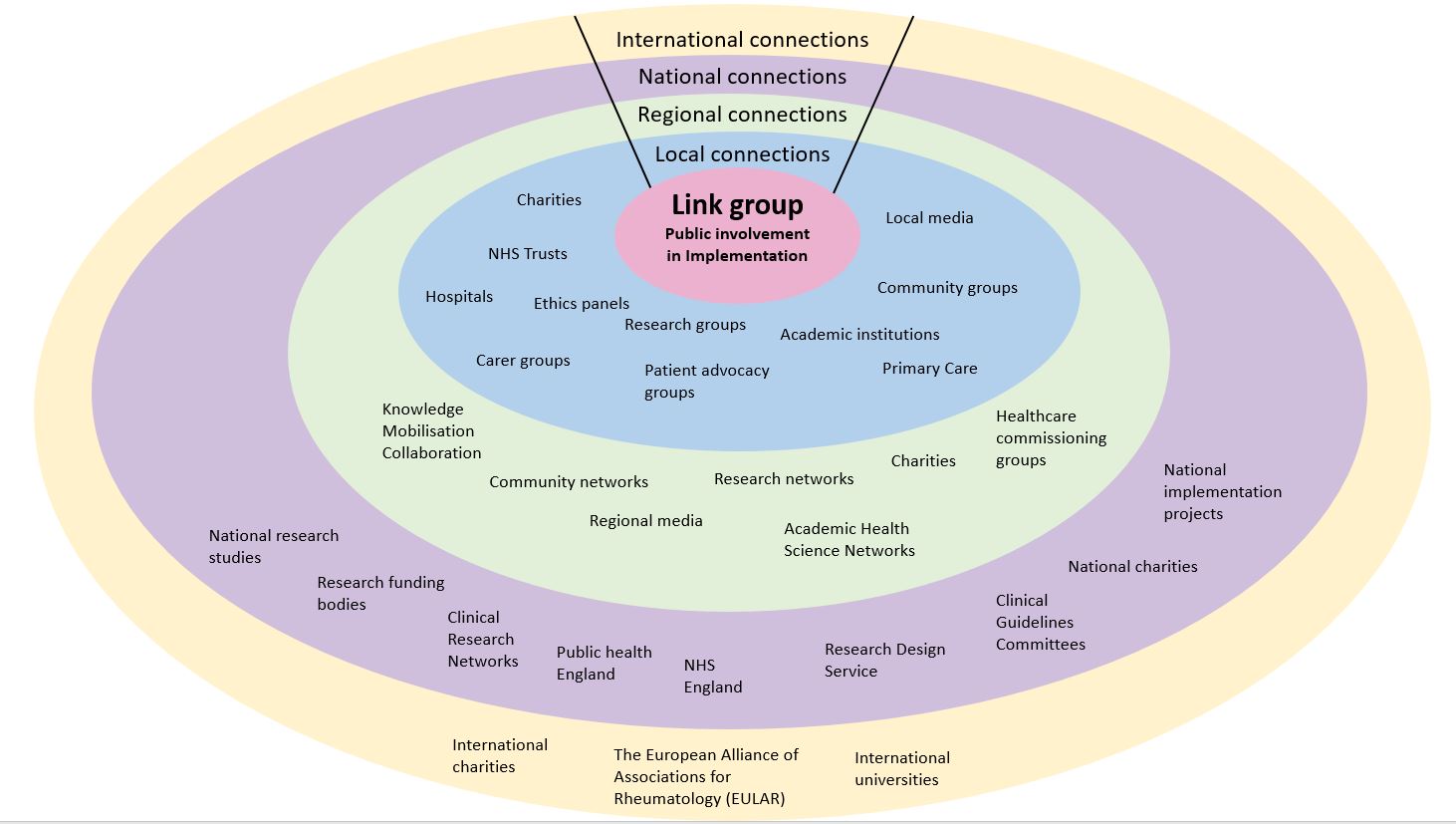

Supplement: Supplemental Material [file IGEN_A_2243037_SM9145.docx]
